# Abstract of Meteorological Observations for July, 1855, Made at Philadelphia, Pa.

**Published:** 1855-09

**Authors:** James A. Kirkpatrick


*Abstract of Meteorological Observations for July,* 1855, *made at Philadelphia, Pa. Latitude* 39° 57′ 28″ *N., Longitude* 75° 10′ 40″ *W. from Greenwich.* By PROF. JAMES A. KIRKPATRICK.

| 1855. July. | Barometer. Daily Mean | Barometer. Mean Daily Range. | Thermom. Daily Mean | Thermom. Mean Daily Range | Dew Point 2 P.M. | Force of Vapor 2 P.M. | Rel. Humid. 2 P.M. | Rain. | Prevailing Winds. | Remarks. |
|---|---|---|---|---|---|---|---|---|---|---|
| | Inches. | Inches. | Deg. | Deg. | Deg. | Inches. | Hunds. | Inch. | Points. | |
| 1 | 29.739 | .036 | 85.7 | 3.0 | 70.6 | .749 | .53 | | SSW. | Clear; ev. heat lightning. |
| 2 | 29.770 | .058 | 84.3 | 2.0 | 67.0 | .662 | .52 | | SW. | M. and aft. clear; ev. cloudy. |
| 3 | 29.950 | .180 | 82.3 | 2.0 | 62.7 | .570 | .47 | | (Var.) | Cloudy. |
| 4 | 29.853 | .097 | 80.7 | 1.7 | 68.2 | .689 | .57 | 0.145 | SW. | Cloudy; aft. and ev. *rain*, |
| 5 | 29.884 | .058 | 80.3 | 2.3 | 64.0 | .597 | .53 | | (Var.) | M. cloudy; aft. and ev. clear. |
| 6 | 29.825 | .070 | 78.0 | 2.3 | 73.6 | .828 | .78 | 0.165 | (Var.) | Cl'y; *showery;* th'r & l'ng. |
| 7 | 29.742 | .109 | 69.0 | 9.0 | 63.8 | .591 | .89 | 1.353 | NW. | Cloudy; m. and aft. *rain.* |
| 8 | 29.965 | .224 | 70 5 | 8.2 | 52.4 | .395 | .43 | | NNW. | Clear. |
| 9 | 30.037 | .071 | 73.3 | 3.8 | 58.8 | .497 | .45 | | (Var.) | M. clear; aft. and ev. cloudy. |
| 10 | 29.883 | .151 | 75.3 | 3.3 | 68.8 | .703 | .73 | | SW. | M. and ev. clear; aft. cloudy. |
| 11 | 29.761 | .122 | 79.3 | 5.7 | 69.3 | .716 | .64 | | SW. | M. and aft. cloudy; ev. clear. |
| 12 | 29.743 | .018 | 81.3 | 2.0 | 63.9 | .594 | .48 | | W. | Clear. |
| 13 | 29.685 | .058 | 80.8 | 1.8 | 72.1 | .788 | .68 | | SW. | Cloudy. *Bar. lowest* 29.645. |
| 14 | 29.828 | .143 | 79.8 | 1.0 | 61.4 | .544 | .47 | | NW. | Clear. |
| 15 | 30.083 | .255 | 78.5 | 2.0 | 64.6 | .608 | .51 | | NE. | Cloudy. *Bar. highest* 30.135. |
| 16 | 30.107 | .048 | 80.8 | 2.3 | 68.8 | .704 | .59 | | W. | M. hazy; aft. & ev. cloudy. |
| 17 | 29.990 | .116 | 83.7 | 2.8 | 73.4 | .821 | .62 | | SW. | M. cloudy; aft. and ev. clear. |
| 18 | 29.863 | .128 | 86.7 | 3.0 | 75.2 | .873 | .61 | | W. | M. and aft. cloudy; ev. clear. |
| 19 | 29.755 | .108 | 88.0 | 2.0 | 73.2 | .818 | .50 | | W. | Clear. *Th. highest* 95° |
| 20 | 29.755 | .054 | 84.3 | 4.3 | 74.2 | .845 | .54 | 2.835 | (Var.) | Cloudy; ev. *rain*, th'r & l'ng. |
| 21 | 29.852 | .097 | 67.8 | 16.5 | 61.7 | .550 | .82 | 0.608 | NE. | Cl'y; *driz'ng & raining* all day |
| 22 | 29.958 | .106 | 64.2 | 6.0 | 62.8 | .570 | .89 | 0.330 | NE. | Cl'y; m. & a. *rain. Th. l'st* 57° |
| 23 | 29.954 | .019 | 72.3 | 8.2 | 68.3 | .692 | .77 | | (Var.) | Cloudy. |
| 24 | 29.900 | .055 | 80.8 | 8.5 | 76.4 | .908 | .75 | 0.167 | SE. | Cl'dy; *showery;* ev. heat l'ng. |
| 25 | 29.853 | .047 | 83.7 | 2.8 | 78 5 | .975 | .76 | | WSW. | Cloudy. |
| 26 | 29.792 | .061 | 85.3 | 1.7 | 75.2 | .872 | .60 | 0.248 | SW. | Cl'y; m. & ev. *rain*, with l'ng. |
| 27 | 29.740 | .052 | 85.0 | 1.0 | 73.6 | .829 | .60 | | SW. | Clear. |
| 28 | 29.741 | .010 | 82.3 | 2.7 | 73.9 | .835 | .65 | | NNE. | M. cloudy; aft. and ev. clear. |
| 29 | 29.787 | .046 | 84.8 | 2.5 | 71.2 | .763 | .56 | | NW. | M. cl'y; aft. & ev. cl'r. [*rain.* |
| 30 | 29.780 | .025 | 80.5 | 4.7 | 69.3 | .716 | .64 | 0.743 | (Var.) | Cl'y; 1 P.M. th. & l'ng; 2 to 8 P.M. |
| 31 | 29.799 | .041 | 81.7 | 2.8 | 71.6 | .774 | .64 | | (Var.) | M. cloudy; aft. and ev. clear. |
| Means for July, 1855. | 29.851 | .086 | 79.7 | 3.9 | 68.7 | .712 | .62 | 6.594 | S. 68° W., 43-100. | |
| Means for July, 5 yrs. | 29.878 | .082 | 77.6 | 3.7 | 59.4 | | | 5.780 | S. 73½° W., 17-100. | |

The Monthly Range of the Barometer was 0.490 of an inch, and of the Thermometer 38°.

